# Peroxiredoxin 1-Toll-like receptor 4-p65 axis inhibits receptor activator of nuclear factor kappa-B ligand-mediated osteoclast differentiation

**DOI:** 10.1016/j.isci.2024.111455

**Published:** 2024-11-26

**Authors:** Jisu Park, Sanggil Kim, Hye-Yeon Jung, Eun Hwan Bae, Minhye Shin, Jae-Il Park, So-Young Choi, Sun-Ju Yi, Kyunghwan Kim

**Affiliations:** 1 Department of Biological Sciences and Biotechnology, Chungbuk National University, Cheongju, Chungbuk, Republic of Korea; 2 Department of Lead Optimization, New Drug Development Center, Osong Medical Innovation Foundation (KBio), 123 Osongsaengmyeng-ro, Cheongju, Chungbuk, Republic of Korea; 3 Korea Basic Science Institute, Gwangju Center at Chonnam National University, Gwangju, Republic of Korea; 4 Department of Microbiology, College of Medicine, Inha University, Incheon, Republic of Korea

**Keywords:** Cellular physiology, Molecular biology, Transcriptomics

## Abstract

Peroxiredoxin 1 (PRDX1), an intracellular antioxidant enzyme, has emerged as a regulator of inflammatory responses via Toll-like receptor 4 (TLR4) signaling. Despite this, the mechanistic details of the PRDX1-TLR4 axis and its impact on osteoclast differentiation remain elusive. Here, we show that PRDX1 suppresses RANKL-induced osteoclast differentiation. Utilizing pharmacological inhibitors, we reveal that PRDX1 inhibits osteoclastogenesis through both TLR4/TRIF and TLR4/MyD88 pathways. Transcriptome analysis revealed PRDX1-mediated alterations in gene expression, particularly upregulating serum amyloid A3 (*Saa3*) and aconitate decarboxylase 1 (*Acod1*). Mechanistically, PRDX1-TLR4 signaling activates p65, promoting *Saa3* and *Acod1* expression while inhibiting *Nfatc1*, a master regulator of osteoclastogenesis. Remarkably, PRDX1 redirects p65 binding from *Nfatc1* to *Saa3* and *Acod1* promoters, thereby suppressing osteoclast formation. Structural analysis showed that a monomeric PRDX1 mutant with enhanced TLR4 binding exhibited the potent inhibition of osteoclast differentiation. These findings reveal the PRDX1-TLR4 axis’s role in inhibiting osteoclastogenesis, offering potential therapeutic insights for bone disorders.

## Highlights

• PRDX1 suppresses RANKL-induced osteoclast differentiation through TLR4 signaling
• PRDX1-TLR4 signaling upregulates *Saa3* and *Acod1*, inhibiting osteoclastogenic genes
• PRDX1 redirects p65 from Nfatc1 to *Saa3* and *Acod1* promoters
• A monomeric PRDX1 mutant enhances TLR4 binding, boosting anti-osteoclast effects

## Graphical abstract

**Figure:**
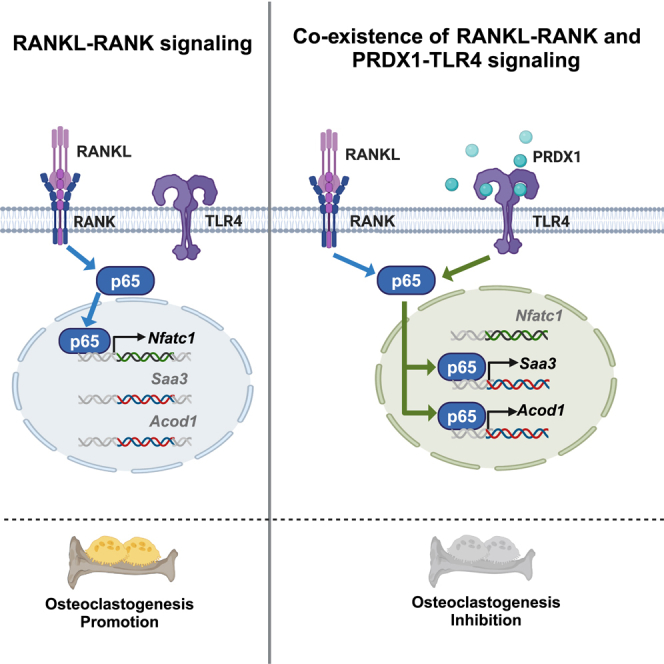

## Introduction

Throughout life, healthy bones undergo constant remodeling. This process involves the coordinated actions of two cell types: osteoblasts, which are responsible for bone formation, and osteoclasts, which are involved in bone resorption. This balance is essential for bone health. However, when the activity of osteoclasts surpasses that of osteoblasts, bone is excessively broken down, leading to osteoporosis.^1^^,^^2^^,^^3^^,^^4^^,^^5^

Osteoclasts are differentiated from monocytes or osteoclast precursor (OCP) cells, which originate from hematopoietic stem cells. Two well-known factors, macrophage colony stimulating factor (M-CSF) and receptor activator of nuclear factor kappa-B ligand (RANKL) play a key role in the osteoclast differentiation.^6^^,^^7^ RANKL binds to its receptor, the receptor activator of nuclear factor kappa-B (RANK), which activates several transcription factors, such as c-Fos and nuclear factor kappa-B(NF-κB).^8^^,^^9^^,^^10^ These transcription factors translocate to the nucleus and promote expression of a set of critical osteoclast-specific genes such as nuclear factor-activated T cells c1 (NFATc1), tartrate-resistant acid phosphatase (TRAP), cathepsin K, and matrix metalloproteinase-9 (MMP-9). Especially, NFATc1 is known as the master regulator of osteoclastogenesis and upregulates a variety of genes responsible for osteoclast adhesion, migration, acidification, degradation of bone matrix such as TRAP, cathepsin K, calcitonin receptor, and osteoclast-associated receptor (OSCAR).^11^^,^^12^^,^^13^^,^^14^^,^^15^^,^^16^

Peroxiredoxin 1 (PRDX1) is a member of the PRDX family, functioning as a cysteine-dependent peroxidase that reduces reactive oxygen species (ROS).^17^^,^^18^^,^^19^ Widely expressed in tissues, PRDX1 serves as an intracellular antioxidant enzyme, implicated in various cellular processes including tumor suppression, apoptosis, and chaperone function.^20^^,^^21^^,^^22^^,^^23^^,^^24^^,^^25^^,^^26^^,^^27^ Previous studies by Neumann et al. have demonstrated that Prdx1-knockout mice, while viable and fertile, develop severe hemolytic anemia and exhibit an increased incidence of malignant cancers.^27^ The tumor suppressive role of PRDX1 has been particularly evident in breast and lung cancer models using these knockout mice.^25^^,^^28^ Additionally, elevated nuclear ROS levels in primary tissues isolated from another Prdx1-knockout mouse model resulted in increased DNA damage and heightened cancer susceptibility.^26^ PRDX1’s pathological roles extend to cardiovascular diseases,^29^^,^^30^^,^^31^ neurodegenerative disorders,^32^ and inflammation.^33^^,^^34^ As key players in inflammatory processes, peroxiredoxins serve multiple functions: they act as cytoprotective enzymes against elevated ROS/RNS levels generated during inflammation, modulate redox signaling to regulate essential functions in inflammatory cells, and can serve as extracellular pathogen-associated molecular patterns (PAMPs) or damage-associated molecule.^35^^,^^36^ Recent findings indicate that PRDX1 is extracellularly released and binds to toll-like receptor 4 (TLR4), triggering the secretion of proinflammatory cytokines.^21^^,^^37^ Interestingly, the structural variants of PRDX1 influence its binding affinity with TLR4, with mutants exhibiting differing effects on interleukin-6 (IL-6) levels. The PRDX1 C52S mutant, which lacks peroxidase activity but is capable of forming decamer, elicited IL-6 levels comparable to those induced by wild type PRDX1. Conversely, the PRDX1 C83S mutant, which retains peroxidase activity but is unable to form a decamer, resulted in a smaller increase in IL-6 levels compared to wild type PRDX1.^38^^,^^39^

In the context of RANKL-induced osteoclast differentiation, TLR4 ligands have been reported to modulate osteoclastogenesis. For example, the treatment of osteoclast precursors with haptoglobin in the presence of M-CSF and RANKL prevents osteoclastogenesis via the haptoglobin-TLR4-interferon-β axis.^40^ Various TLR stimulations, such as peptidoglycan, dsRNA, LPS, and CpG DNA motifs, also suppress osteoclast differentiation.^41^ Among them, LPS, a known TLR4 ligand, modulates osteoclast differentiation depending on the timing of its addition during RANKL-induced osteoclastogenesis. LPS inhibits the differentiation of osteoclast precursors into osteoclast but increases osteoclast formation in RANKL-primed cells.^42^^,^^43^

These findings prompted us to investigate whether extracellular PRDX1 could regulate RANKL-mediated osteoclast differentiation. Our study unveiled the pivotal role of the PRDX1-TLR4 axis in inhibiting osteoclast precursor differentiation into osteoclasts. Transcriptome analysis revealed differential gene expression upon PRDX1 treatment, notably upregulating *Saa3* and *Acod1* expression, which are associated with reduced osteoclast differentiation or ATP levels, respectively. PRDX1 activated NF-κB p65 through both TLR4-MyD88 and TLR4-TRIF signaling pathways, enriching it on the promoters of *Saa3* and *Acod1*. Additionally, PRDX1 inhibited NFATc1 expression and p65 localization on the NFATc1 promoter, collectively demonstrating the modulation of p65 enrichment on specific gene promoters by the PRDX1-TLR4 axis, thereby blocking RANKL-induced osteoclast differentiation.

## Results

### Extracellular peroxiredoxin 1 suppresses receptor activator of nuclear factor kappa-B ligand-induced osteoclastogenesis through Toll-like receptor 4 signaling

**Figure 1:**
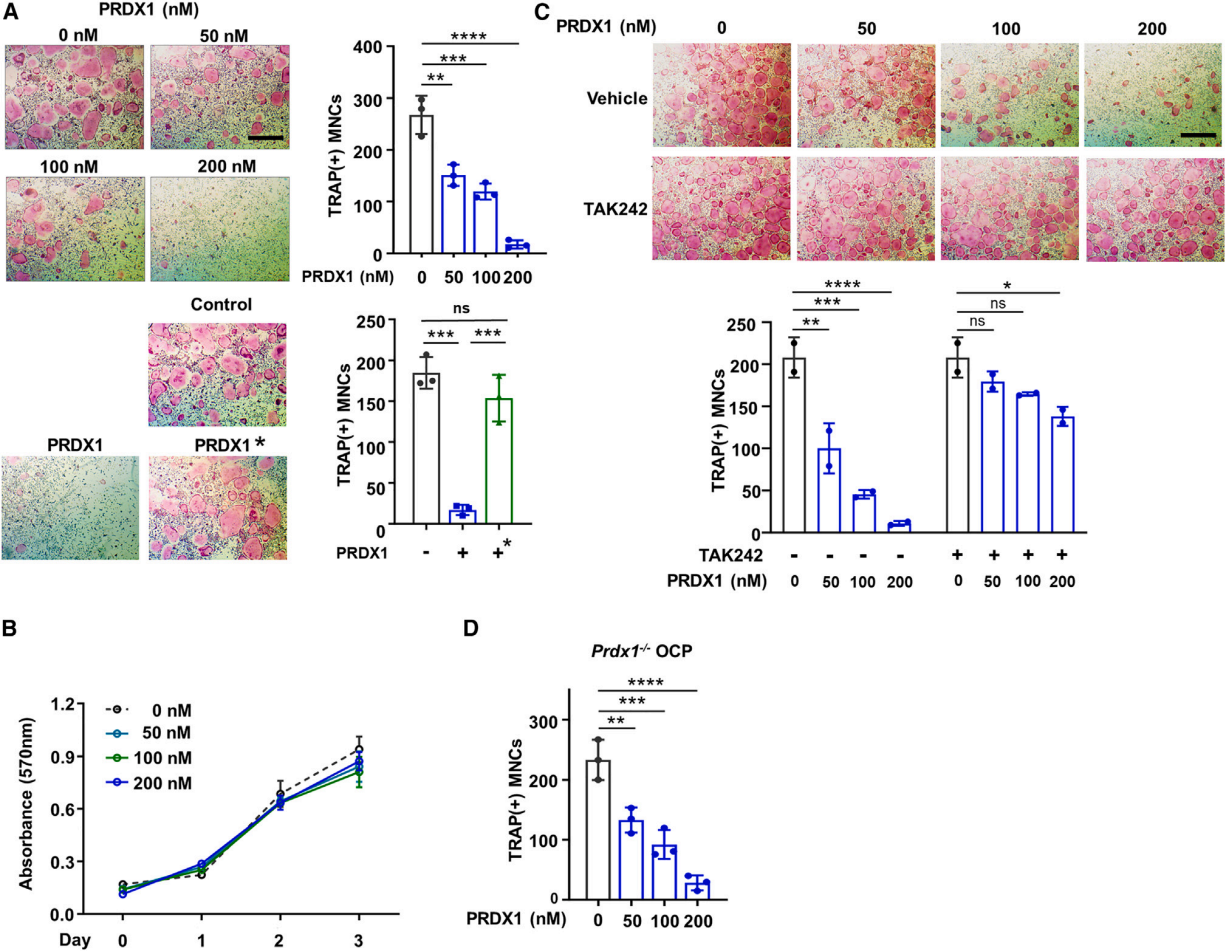
Effects of extracellular peroxiredoxin 1 (PRDX1) on osteoclast differentiation (A) Osteoclast precursor (OCP) cells were cultured in the presence of M-CSF (30 ng/mL), and RANKL (100 ng/mL) with various concentrations of recombinant PRDX1. Heat-inactivated PRDX1 (marked with an asterisk) was heated at 95°C for 30 min before treatment. Osteoclasts were stained with tartrate-resistant acid phosphates (TRAP) and TRAP-positive multinucleated cells were counted. Scale bar, 75μm. (B) OCP cells were cultured with various concentrations of PRDX1 in the presence of M-CSF (30 ng/mL). Cell proliferations were measured for a period of 3 days. (C) OCP cells were cultured with various concentrations of PRDX1 in the absence or presence of TAK242 (5 μM) during osteoclastogenesis. TRAP-positive multinucleated cells were counted. Scale bar, 75μm. (D) OCP cells derived from *Prdx1*^*−/−*^ mice were treated with M-CSF (30 ng/mL) and RANKL (100 ng/mL) in the presence of PRDX1 as indicated. TRAP-positive multinucleated cells were counted. The data are presented as the mean ± SD values from three independent experiments; P-value was examined by one-way ANOVA with Tukey’s multiple comparisons test. ∗*p* < 0.05; ∗∗*p* < 0.01; ∗∗∗*p* < 0.001; ∗∗∗∗*p* < 0.0001.

To study the effect of extracellular PRDX1 in osteoclastogenesis, we performed the osteoclast differentiation assays using purified recombinant PRDX1 protein. Our findings showed that extracellular PRDX1 significantly suppressed osteoclast formation in a dose-dependent manner (Figures 1A and S1). When we tested the same concentration of heat-inactivated PRDX1, no inhibition of osteoclast formation was observed, reinforcing the specificity of PRDX1’s repressive effect on osteoclast formation. Notably, the finding that PRDX1 treatment did not influence cell proliferation suggests that PRDX1 selectively impedes the differentiation of OCP cells without interfering with their proliferation (Figure 1B).

Given that extracellular PRDX1 stimulates inflammatory responses via TLR4 binding,^21^^,^^38^ we next examined whether PRDX1-TLR4 signaling could interfere with RANKL-mediated osteoclast differentiation. To do this, we employed TAK242, a TLR4-specific inhibitor that blocks further TLR4 signaling. Co-treatment with PRDX1 and TAK242 eliminated PRDX1’s inhibitory effect (Figure 1C), indicating that Prdx1-TLR4 signaling plays a key role in hindering osteoclast differentiation. Our studies have demonstrated that intracellular Prdx1 suppresses RANKL-mediated osteoclast differentiation by scavenging reactive oxygen species (unpublished data). To assess whether PRDX1-TLR4 signaling depends on intracellular PRDX1 enzymatic activity, we used OCP cells from *Prdx1* knockout mice (Figure 1D). Despite the absence of intracellular PRDX1, osteoclast differentiation was still reduced following extracellular PRDX1 treatment. These findings suggested that extracellular PRDX1 clearly inhibits RANKL-induced osteoclast differentiation independently of intracellular PRDX1.

### Peroxiredoxin 1-Toll-like receptor 4 signaling alters diverse gene expression to inhibit osteoclast differentiation

**Figure 2:**
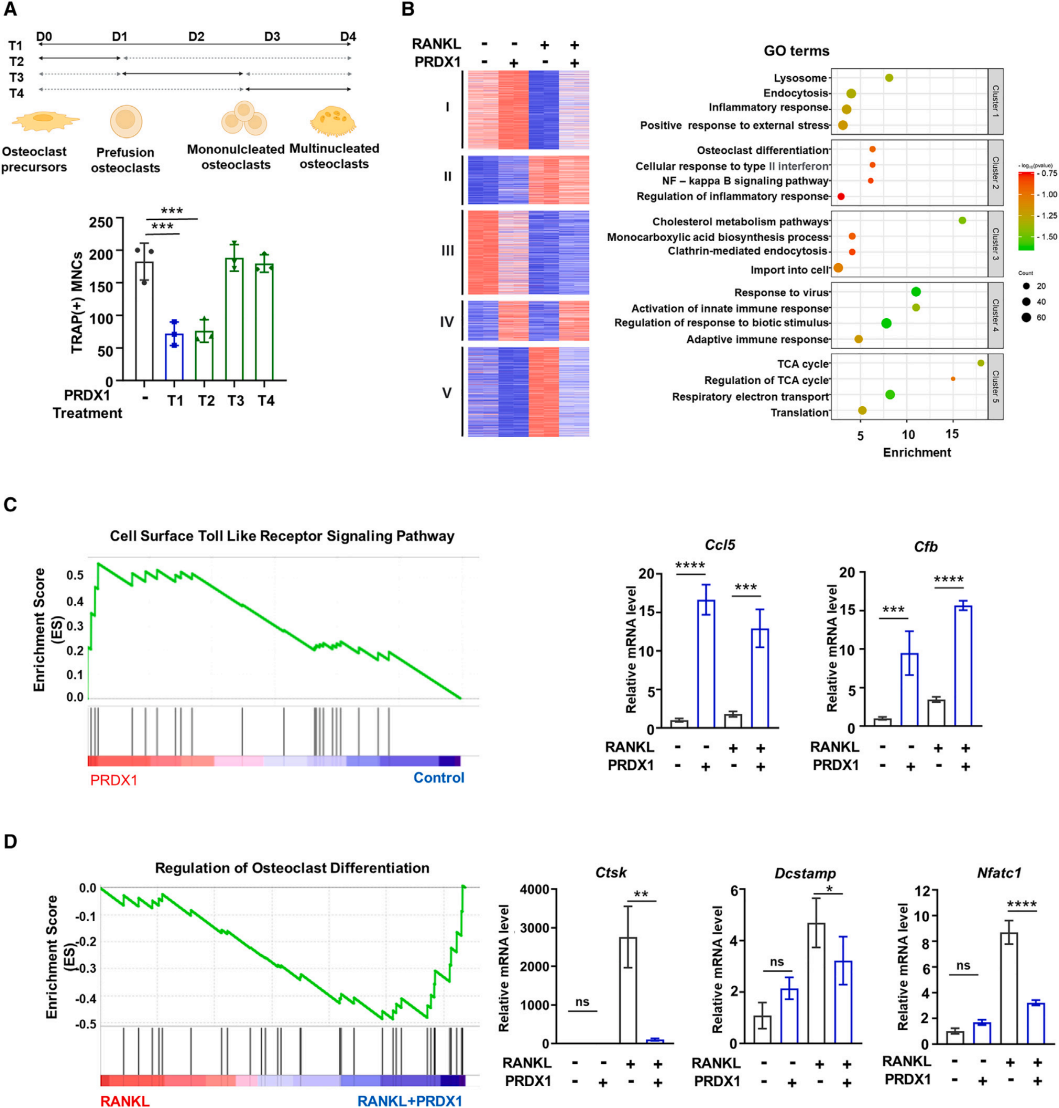
Gene profiling analysis of PRDX1 during osteoclastogenesis (A) The period for osteoclast differentiation was divided into four distinct time periods (T1 to T4). PRDX1 (200 nM) was treated during each period, and TRAP-positive multinucleated cells were counted. (B) Heatmap shows mRNA expression profiles of PRDX1 during osteoclastogenesis. K-means (K = 5) clustering of 4,328 differentially expressed genes (DEGs) in any pairwise comparison among four conditions (left panel). The clusters are indicated on the left. The right panel shows a heatmap showing the P-value significance and gene numbers of gene ontology (GO) analysis for genes in each cluster. (C) GSEA plot shows the increased gene expression of cell surface toll-like receptor signaling pathway in PRDX1 (PRDX1 + no RANKL condition) compared to control (no PRDX1 + no RANKL). *Cfb* and *Ccl5* expression levels were measured by RT-qPCR using specific primers. (D) GSEA plot shows the decreased gene expression of the regulation of osteoclast differentiation in PRDX1 + RANKL treatment compared to RANKL treatment. mRNA expression levels of *Ctsk*, *Dcstamp*, and *Nfatc1* were measured by RT-qPCR. The data are presented as the mean ± SD values from three independent experiments; P-value was examined by one-way ANOVA with Tukey’s multiple comparisons test. ∗*p* < 0.05; ∗∗*p* < 0.01; ∗∗∗*p* < 0.001; ∗∗∗∗*p* < 0.0001.

Osteoclast differentiation involves several stages,^44^ of which we have divided into three distinct phases (Figure 2A): pre-osteoclasts (pOCs), mononucleated osteoclasts, and multinucleated osteoclasts. To determine the timing of PRDX1 treatment that affects osteoclast differentiation, we applied PRDX1 throughout the entire differentiation process (T1) and during three specific periods (T2, T3, and T4). Our results indicated that PRDX1 primarily influences the early stage (T2) of osteoclast differentiation (Figure 2A). To further investigate which genes are regulated by PRDX1-TLR4 signaling, we performed RNA sequencing using mRNA from samples treated with PRDX1 in the presence and absence of RANKL for two days. We analyzed four treatment groups: 1) no PRDX1 + no RANKL, 2) PRDX1 + no RANKL, 3) no PRDX1 + RANKL, and 4) PRDX1 + RANKL. Genome-wide transcriptome analysis identified a set of differentially expressed genes in pairwise comparisons across the four conditions. K-means clustering revealed five gene clusters that were differentially influenced by PRDX1 and/or RANKL treatment (Table S1). We analyzed gene function in each cluster using gene ontology (GO) term analysis (Figure 2B). Given our findings that PRDX1 acts as a ligand inducing gene expression and that it inhibits RANKL-induced osteoclast formation, we focused primarily on clusters 4 (upregulated by Prdx1 treatment) and 5 (RANKL-induced genes downregulated by PRDX1 treatment). Cluster 4 showed genes enriched in proinflammatory responses associated with the TLR4 signaling pathway, reinforcing our and others’ findings that PRDX1 signaling is closely linked to TLR4 signaling (Figures 2B and S2A). On the other hand, cluster 5 featured genes related to TCA cycle components, suggesting that PRDX1 signaling may regulate these components to inhibit RANKL-mediated osteoclast differentiation (Figures 2B and S2B). To further validate our findings, we conducted gene set enrichment analysis (GSEA), which showed a positive correlation with the cell surface TLR signaling pathway when comparing 1) no PRDX1 + no RANKL and 2) PRDX1 + no RANKL conditions (Figure 2C). Notably, we identified increased expression of *Cfb* and *Ccl5*, both marker genes of TLR4 signaling^45^^,^^46^ (Figure 2C), indicating a strong link to the TLR4 pathway. In contrast, comparing 3) no PRDX1 + RANKL and 4) PRDX1 + RANKL conditions showed a negative correlation with osteoclast differentiation (Figure 2D). We also observed a decrease in both mRNA and protein levels of the *Ctsk*, *Dcstamp,* and *Nfatc1* genes following PRDX1 treatment (Figure 2D; Figure S2C).

### Peroxiredoxin 1-Toll-like receptor 4 positively regulates serum amyloid A3 and aconitate decarboxylase 1 to inhibit osteoclast differentiation

**Figure 3:**
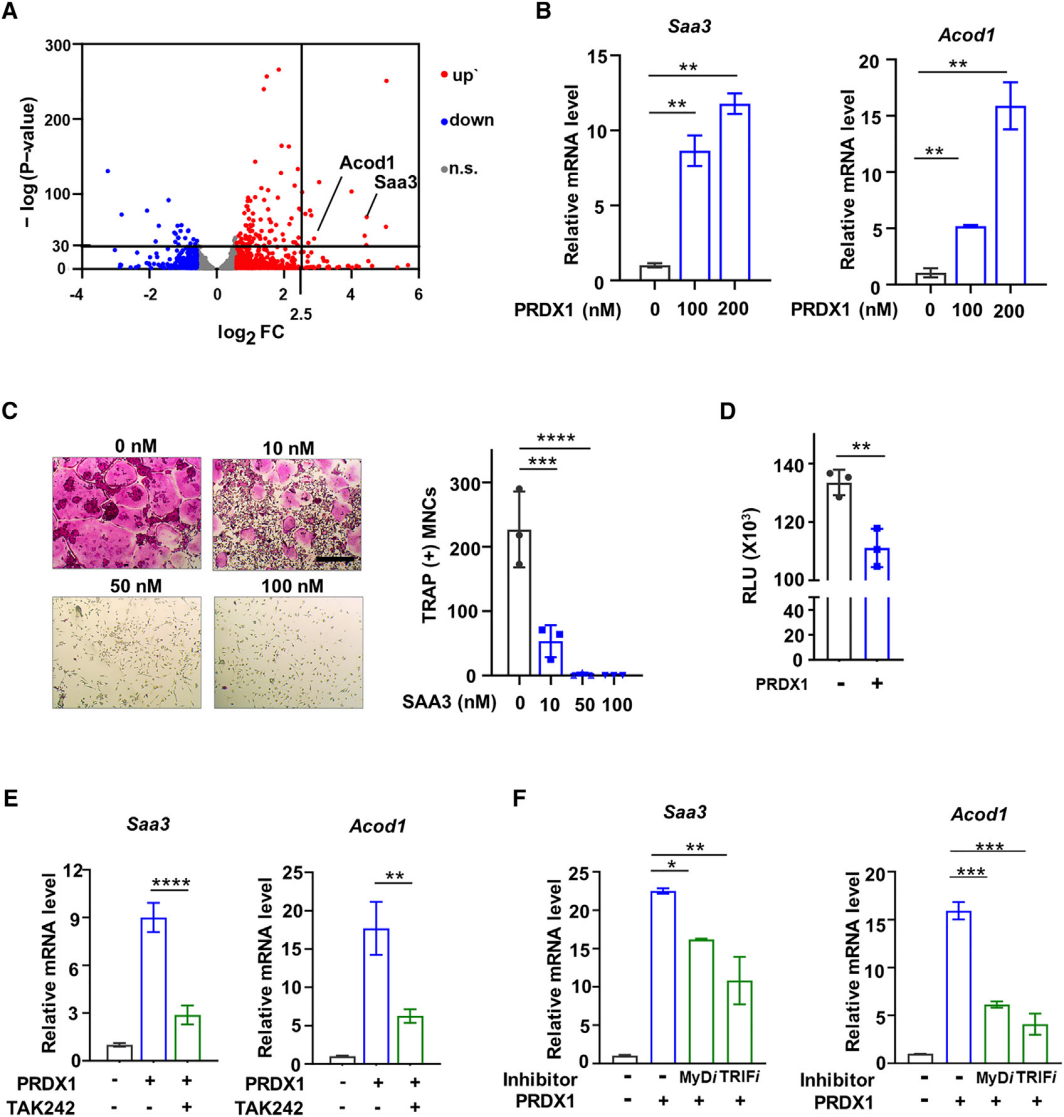
PRDX1 induces expressions of *Saa3* and *Acod1* via TLR4 to inhibit osteoclast differentiation (A) Volcano plot of transcriptomic changes in PRDX1 (PRDX1 + no RANKL condition) compared to control (no PRDX1 + no RANKL). Colored dots correspond to genes with significant (FDR <0.05) and greater than 1.5-fold expression changes. Baselines indicate genes with substantial (-log FDR >30) and greater than 2.5-fold expression changes. (B) OCP cells were treated with M-CSF (30 ng/mL) in the presence or absence of PRDX1 for 1 day. *Saa3* and *Acod1* mRNA expressions were measured by RT-qPCR. (C) OCP cells were incubated for 4 days with SAA3 at various concentrations in the presence of M-CSF (30 ng/mL) and RANKL (100 ng/mL). TRAP staining was performed for the counting of multinucleated cells. Scale bar, 75μm. (D) OCP cells were incubated for 2 days with PRDX1 (200 nM) in the presence of RANKL (100 ng/mL). Cellular ATP levels were measured. (E) *Saa3* and *Acod1* expression with TAK242 (5 μM) in the presence of PRDX1 (200 nM) for 1 day was measured by RT-qPCR. (F) *Saa3* and *Acod1* expression with MyD or TRIF inhibitor (each 5 μM) in the presence of PRDX1 (200 nM) for 1 day was measured by RT-qPCR. The results were reported as Mean ± SD from three independent experiments. *p* value is determined by a two-tailed *t*-test in (D) and one-way ANOVA with Tukey’s multiple comparisons test in (B, C, E, and, F). ∗*p* < 0.05; ∗∗*p* < 0.01; ∗∗∗*p* < 0.001; ∗∗∗∗*p* < 0.0001.

Next, we questioned which target genes of the PRDX1-TLR4 signaling pathways regulate RANKL signaling pathways. Based on cluster 4 and a volcano plot (upregulated genes in 1) no PRDX1 + no RANKL versus 2) PRDX1 + no RANKL), we identified *Saa3* and *Acod1* as potential target genes (Figure 3A; Table S2). To investigate whether PRDX1 influences the mRNA expression of *Saa3* and *Acod1*, we performed RT-qPCR, confirming that PRDX1 induces the expression of *Saa3* and *Acod1* at the early stage of osteoclast differentiation (Figure 3B). The SAA family, particularly SAA1, has been shown to inhibit osteoclast differentiation as extracellular ligands,^47^^,^^48^ raising the question of whether SAA3, another member of the SAA family, inhibits osteoclast differentiation. we then examined the effect of SAA3 on osteoclast formation. As expected, the treatment of recombinant SAA3 effectively inhibits osteoclast formation (Figure 3C). Additionally, ACOD1, which is upregulated by inflammatory stimuli, produces itaconate from *cis*-aconitate, an intermediate of the TCA cycle, in macrophages.^49^ The increased itaconate inhibits succinate dehydrogenase which is an essential component for the TCA cycle and cellular respiration via the electron transport chain.^50^ Furthermore, ACOD1 plays a significant role in reducing ATP production in response to LPS.^51^ Given recent studies demonstrating the negative regulation of osteoclastogenesis by ACOD1 and itaconate^52^^,^^53^^,^^54^ and our findings showing reduced expression of genes related to the TCA cycle and respiratory electron transport (Figure 2B), we investigated whether PRDX1 influences ATP production, a critical factor for osteoclast fusion and differentiation. To do this, we measured ATP concentrations following PRDX1 treatment. The result showed that PRDX1 treatment significantly decreased ATP levels (Figure 3D). This suggests a potential link between PRDX1-induced expression of *Acod1* and reduced intracellular ATP levels, leading to the inhibition of RANKL-induced osteoclast differentiation.

To assess whether binding PRDX1 to TLR4 directly influences *Saa3* and *Acod1* gene expression, we used a TAK242 inhibitor. Treatment with TAK242 significantly reduced the expression of *Saa3* or *Acod1* induced by PRDX1 (Figure 3E). This suggests that PRDX1’s binding to TLR4 triggers the expression of *Saa3* and *Acod1*. TLR4 signaling encompasses two pathways: the MyD88-dependent and TRIF-dependent pathways.^55^ Utilizing inhibitors for each, we found that PRDX1-TLR4 signaling employs both pathways for the expression of these genes (Figure 3F).

### PRDX1 modulates the transcription factor p65 signaling pathway to stimulate the expression of *Saa3* and *Acod1*

**Figure 4:**
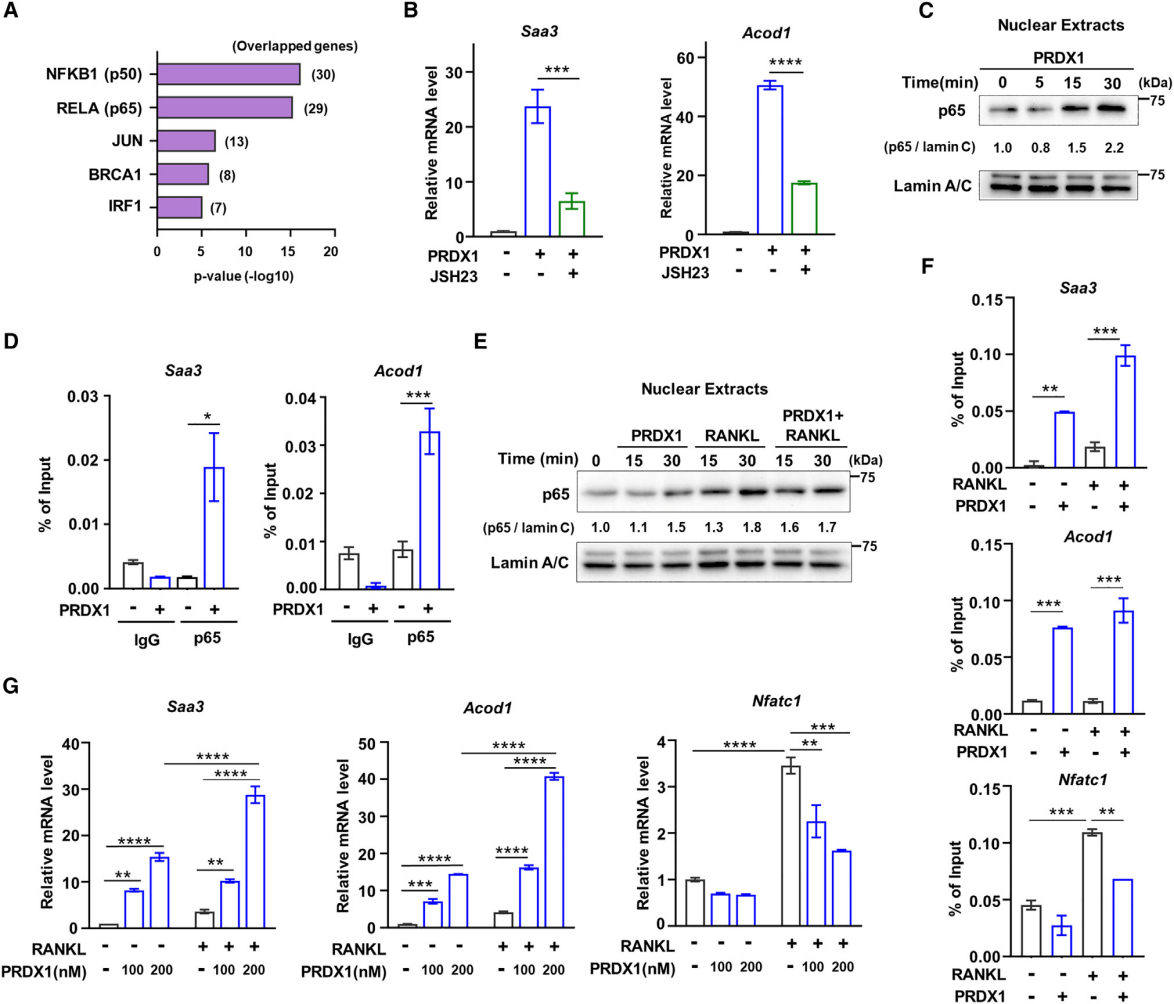
p65 is the transcription factor for PRDX1-TLR4 signaling (A) The most significantly enriched transcription factors regulating PRDX1 target genes belonging to cluster 4 (Figure 2B) using TRRUST v2. (B) *Saa3* and *Acod1* expression levels were measured from OCP cells treated with JSH23 (20 μM) in the presence of PRDX1 (200 nM). (C) OCP cells were treated with PRDX1 (200 nM) at the indicated times. Nuclear translocation of p65 was analyzed by immunoblotting with p65 antibody. The immunoblot band was quantified using ImageJ software and normalized to lamin C band intensity. (D) ChIP assay of p65 occupancy at the *Saa3* and *Acod1* promoters after 30 min of PRDX1 (200 nM) treatment. (E) OCP cells were treated with a combination of PRDX1 (200 nM) and RANKL (100 ng/mL) as indicated. Nuclear extracts were analyzed by immunoblotting for p65. The immunoblot band intensity was quantified using ImageJ software and normalized to lamin C band intensity. (F) ChIP assay of p65 occupancy at the *Saa3*, *Acod1*, and *Nfatc1* promoters with a combination of PRDX1 (200 nM) and RANKL (100 ng/mL) for 30 min. (G) mRNA expression levels of *Saa3*, *Acod1*, and *Nfatc1* treated with PRDX1 (100 and 200 nM) and/or RANKL (100 ng/mL) for 1 day. The data were presented as Mean ± SD from three independent experiments. *p* value is determined by a two-tailed *t*-test in (D) and one-way ANOVA with Tukey’s multiple comparisons test in (B, C, E, and, F). ∗*p* < 0.05; ∗∗*p* < 0.01; ∗∗∗*p* < 0.001; ∗∗∗∗*p* < 0.0001.

Since PRDX1-TLR4 positively influences *Saa3* and *Acod1* gene expression through both MyD88-and TRIF-dependent pathways, we aimed to identify transcription factors that are downstream in both pathways. By analyzing the genes in cluster 4 using the TRRUST2 program, we found that NF-κB transcription factors, specifically RELA (p65)-NF-κB1 (p50), were highly ranked (Figure 4A). To confirm whether p65-p50 is the main transcription factor in the PRDX1-TLR4 signaling, we used a p65-specific inhibitor, JSH23, during PRDX1 treatment. This inhibitor blocked the expression of *Saa3* and *Acod1* (Figure 4B), indicating that p65 is the key transcription factor for the PRDX1 regulation of *Saa3* and *Acod1* expression.

We further investigated whether p65 translocates into the nucleus upon PRDX1 treatment. Although the overall protein level of p65 remained stable in the whole-cell extract (Figure S3A), its nuclear levels gradually increased over time (Figure 4C; Figure S3B). These findings suggest that PRDX1 signaling prompts p65 translocation into the nucleus. Moreover, ChIP-qPCR confirmed that PRDX1 guides p65 to bind to the promoters of *Saa3* and *Acod1* (Figure 4D), regulating their expression.

Since both PRDX1 and RANKL utilize p65 as a transcription factor, but with opposite effects on osteoclast differentiation, we questioned how PRDX1 inhibits directly the RANKL-mediated p65 signaling pathway. A comparison of p65 expression levels under three conditions—PRDX1 alone, RANKL alone, and simultaneous PRDX1 and RANKL treatment—revealed no significant change in overall p65 expression across all conditions (Figure S3C). Notably, RANKL induced p65 nuclear translocation slightly faster than PRDX1 (Figure 4E). ChIP results showed that PRDX1 directed p65 to bind to the promoters of *Saa3* and *Acod1*, whereas RANKL directed p65 to the *Nfatc1* promoter. Intriguingly, simultaneous transmission of PRDX1 and RANKL signals resulted in predominant p65 binding to the *Saa3* and *Acod1* promoters rather than the *Nfatc1* promoter (Figure 4F). Furthermore, RT-qPCR following PRDX1 and/or RANKL treatment further confirmed these gene expression patterns. *Saa3* and *Acod1* expression increased after PRDX1 treatment and was more pronounced upon co-treatment with PRDX1 and RANKL, while *Nfatc1* expression decreased after co-treatment (Figure 4G). Based on these results, we propose a hijacking model wherein PRDX1 redirects p65 binding from the *Nfatc1* promoter, crucial for osteoclast differentiation, to the promoters of *Saa3* and *Acod1*, thereby inhibiting osteoclastogenesis.

### Peroxiredoxin 1 conformation modulates its interaction with Toll-like receptor 4 and influences osteoclast formation

**Figure 5:**
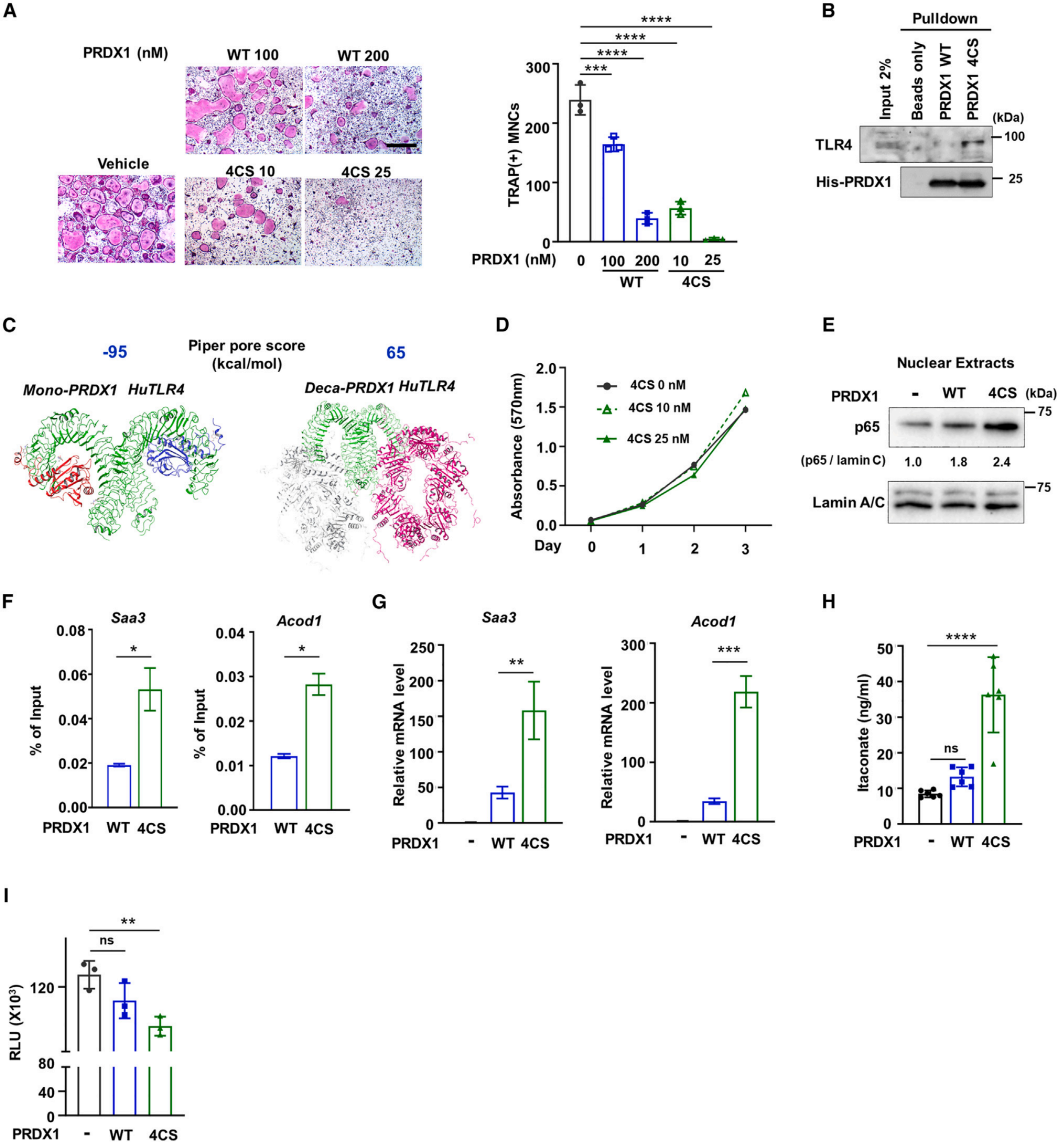
Structural impact of PRDX1 on TLR4 signaling and osteoclast differentiation (A) OCP cells were cultured with varying concentrations of PRDX1 WT (100 and 200 nM) or PRDX1 4CS (10 and 25 nM) in the presence of M-CSF (30 ng/mL) and RANKL (100 ng/mL), followed by TRAP staining. Scale bar, 75μm. (B) Pulldown assay of PRDX1 WT or 4CS with TLR4. His-tagged PRDX1 was used for binding to TLR4. (C) Docking model between human TLR4 (HuTLR4) and PRDX1 mutants. The PIPER Pose Score of the top-scoring entry obtained for the PRDX1-huTLR4 docked complex was determined using the BioLuminate module, utilizing the PIPER program for protein–protein docking. (D) Effect of PRDX1 4CS on cell proliferation. OCP cells were cultured for 3 days with the indicated concentrations of PRDX1 4CS. (E) OCP cells were treated with PRDX1 WT (100 nM) or 4CS mutant (25 nM) in the presence of RANKL (100 ng/mL) for 30 min. Nuclear extracts were analyzed by immunoblotting with p65 antibody. The immunoblot band was quantified using ImageJ software and normalized to lamin C band intensity. (F) ChIP assay of p65 occupancy at the *Saa3*, *Acod1*, and *Nfatc1* promoters treated with PRDX1 WT (100 nM) or 4CS mutant (25 nM) and/or RANKL (100 ng/mL) for 30 min. (G) *Saa3* and *Acod1* mRNA expression levels treated with PRDX1 WT (100 nM) and 4CS mutant (25 nM) for 1 day. (H) OCP cells were incubated with PRDX1 WT (100 nM) or 4CS (25 nM) for 24 h, and cellular itaconate levels were quantified using GC-MS/MS. (I) OCP cells were cultured for 2 days with PRDX1 WT (100 nM), 4CS mutant (25 nM), and/or RANKL (100 ng/ml). Cellular ATP levels were measured. The results shown are mean ± SD values from three independent experiments; P-value is determined by a two-tailed *t*-test in (F) and one-way ANOVA with Tukey’s multiple comparisons test (A, G, H, and I). ∗*p* < 0.05; ∗∗*p* < 0.01; ∗∗∗*p* < 0.001; ∗∗∗∗*p* < 0.0001.

Recent studies have demonstrated that the decameric form of PRDX1, but not the dimer form, is important for the TLR4 signaling pathway.^38^^,^^39^^,^^56^ To further evaluate the impact of PRDX1 structure on osteoclast formation, we generated several mutants including decamer, dimer, and monomer (Figures S1 and S4A). As expected, the dimer form of PRDX1 (C83S mutant) had less effect on osteoclast differentiation, compared to decameric PRDX1 (wild-type) (Figure S4B). Additionally, our protein-protein docking simulation predicted that the decameric form of PRDX1 has a stronger binding affinity with TLR4 than the dimeric form (Figure S4C). Next, we introduced mutations in four cysteine residues (C52, C71, C83, C173) to serine residues (4CS) and confirmed their predominant existence in a monomeric state (Figure S4A). Surprisingly, the PRDX1 4CS mutant exhibited the stronger inhibition of RANKL-mediated osteoclast differentiation than PRDX1 WT, consistent with its stronger binding affinity with TLR4 compared to the WT form (Figures 5A–5D). Remarkably, the nuclear translocation of p65 was increased in the 4CS PRDX1 mutant compared to WT (Figure 5E; Figure S4D), leading to increased localization at the *Saa3* and *Acod1* promoters (Figure 5F), thereby enhancing the expression of *Saa3* and *Acod1* genes (Figure 5G). Consequently, the PRDX1 4CS mutant exhibited increased the production of itaconate and decreased levels of ATP (Figures 5H and 5I).

## Discussion

Recent studies have brought attention to PRDX1 as a ligand for TLR4, but its impact on osteoclast differentiation remains unclear. We reveal that extracellular PRDX1 inhibits early-stage osteoclastogenesis. While previous studies have shown that TLR4 signaling can impede osteoclast formation during this stage,^40^^,^^57^^,^^58^^,^^59^ the precise molecular mechanisms have not been fully elucidated.

Transcriptome analysis revealed significant alterations in gene expression profiles upon PRDX1 treatment, particularly in genes associated with proinflammatory responses and the TCA cycle. Importantly, we identified SAA3 and ACOD1 as potential target genes of the PRDX1-TLR4 signaling pathway. Our study suggests that PRDX1 positively regulates the expression of *Saa3* and *Acod1*, both of which play crucial roles in inhibiting osteoclast differentiation. These findings provide mechanistic insights into how the PRDX1-TLR4 signaling pathway modulates osteoclastogenesis through the regulation of key target genes. Interestingly, recent work by Kwon et al. demonstrated that haptoglobin-mediated TLR4 activation induces IFN-β expression via IRF7. The enhanced IFN-β suppresses osteoclastogenesis by reducing c-Fos expression.^60^ However, in our transcriptome analysis, PRDX1 did not affect *Ifn-β* and *c-Fos* mRNA expression (data not shown). Additionally, we observed the involvement of p65 in *Saa3* and *Acod1* expression. These results underscore the notion that distinct ligands can trigger different signaling mechanisms and outcomes, even when acting via the same TLR4.

**Figure 6:**
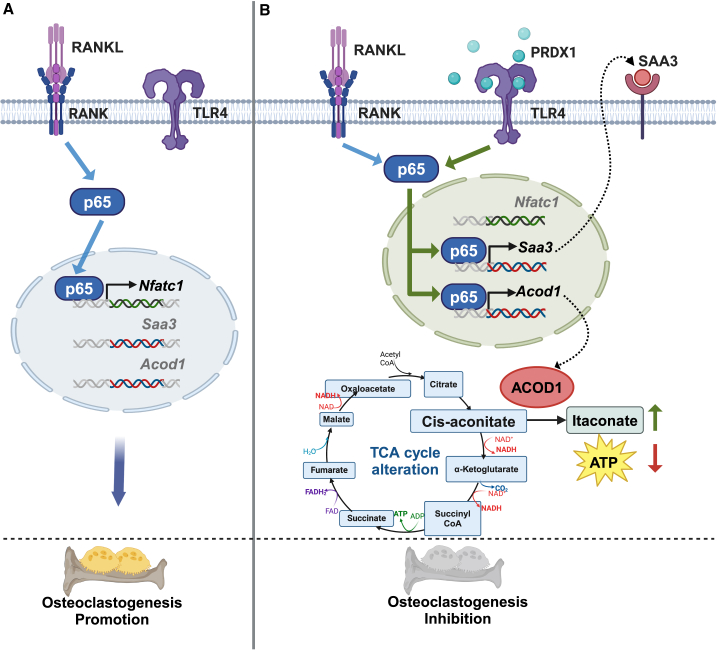
Model represents PRDX1-TLR4 signaling redirecting p65 to the *Saa3* and *Acod1* promoters rather than the *Nfatc1* promoter (A) RANKL-RANK signaling pathway: RANKL binding to RANK activates p65, which translocates to the nucleus and binds to the *Nfatc1* promoter, inducing osteoclastogenesis. (B) PRDX1-TLR4 signaling pathway: Activation of PRDX1-TLR4 signaling redirects p65 to the promoter regions of *Saa3* and *Acod1* instead of *Nfatc1*. As a result, PRDX1 induces the expression of *Saa3* and *Acod1* during RANKL-mediated osteoclast differentiation. The increase in SAA3 and itaconate, produced by ACOD1, inhibits osteoclast differentiation.

In addition to its role in regulating key genes, PRDX1-TLR4 signaling may directly inhibit the RANKL-RANK signaling pathway. The RANKL-RANKL cascade consists of three key steps: (1) binding of RANKL to its receptor RANK, (2) activation of downstream cytosolic pathways such as PI3K-AKT and MAPK, and (3) transcriptional regulation by factors such as p65, c-Fos, and NFATc1 in the nucleus. Our results showed a modest increase in *Rank* mRNA levels following RANKL treatment in the early stage of differentiation (Figure S5); however, PRDX1 did not significantly affect *Rank* mRNA expression, suggesting that PRDX1 does not influence the initial step of the RANKL-RANK pathway. Both the RANKL-RANK and PRDX1-TLR4 pathways converge on the PI3K-AKT and MAPK signaling cascades, complicating efforts to isolate PRDX1’s specific effects on RANKL-induced signaling. Lastly, we explored the influence of PRDX1 on the transcriptional factors involved in RANKL signaling. Interestingly, our study demonstrates that PRDX1-TLR4 signaling downregulates both mRNA and protein levels of *Nfatc1*, a master regulator of osteoclast differentiation controlled by NF-κB and c-Fos (Figure 2D; Figure S2). ChIP analysis further revealed that PRDX1 directs p65 to bind the promoter regions of *Saa3* and *Acod1* instead of *Nfatc1* during osteoclastogenesis. Thus, PRDX1 induces the expression of *Saa3* and *Acod1* rather than *Nfatc1* during RANKL-mediated osteoclast differentiation. This redirection of p65 binding suggests a potential mechanism by which PRDX1 regulates osteoclastogenesis (Figure 6). How would it be possible? One possibility is that the PRDX1-TLR4 pathway could influence the extent of chromatin opening at the other promoters induced by RANKL-RANK signaling. For example, PRDX1-TLR4 signaling may facilitate the opening of chromatin regions at the *Saa3* and *Acod1* promoters, while potentially suppressing chromatin accessibility at the *Nfatc1* promoter. However, our FAIRE-qPCR analysis suggested that chromatin accessibility at the *Saa3* and *Nfatc1* promoters is not affected by PRDX1 and/or RANKL treatment (data not shown). Nevertheless, it is important to note that this assay does not allow for direct comparison of chromatin opening degrees at each promoter. Additionally, other factors such as posttranslational modifications, cofactor binding, and DNA binding affinity could influence p65 ability to determine binding sites. Further study will be needed to explore this aspect.

Moreover, our investigation into the structural variants of PRDX1 revealed intriguing findings. We observed that the decameric form of PRDX1, which has been implicated in the TLR4 signaling pathway, exhibits the stronger inhibition of osteoclast differentiation compared to the dimeric form. Surprisingly, a monomeric form of PRDX1 mutant (4CS) with enhanced binding affinity to TLR4 showed even greater inhibition of osteoclastogenesis. Our results somewhat contradict a recent study.^39^ Liu et al. observed that a monomeric form treated with DTT lost its ligand activity. This discrepancy could be attributed to the susceptibility of the DTT-treated monomer form to oxidation in the oxidized environment (e.g., cell culture media). Alternatively, variations in conformation between the DTT-treated monomeric form and the 4CS mutant could also contribute to the conflicting observations.

In conclusion, our study elucidates the inhibitory role of extracellular PRDX1 in osteoclastogenesis through the TLR4 signaling pathway and provides mechanistic insights into its downstream molecular mechanisms. These findings not only enhance our understanding of the regulatory mechanisms underlying osteoclast differentiation but also have implications for the development of therapeutic strategies for osteoclast-related diseases. Further studies are warranted to explore the therapeutic potential of targeting the PRDX1-TLR4 signaling axis in the treatment of bone-related disorders.

### Limitations of the study

Our study reveals that PRDX1-TLR4 signaling redirects p65 binding from the Nfatc1 promoter to the Saa3 and Acod1 promoters, promoting Saa3 and Acod1 expression while inhibiting Nfatc1. However, the mechanism determining p65’s binding destination remains unclear. Additionally, the effect of PRDX1 in an *in vivo* osteoporosis model was not examined in this study.

## Resource availability

### Lead contact

Further information and requests for resources and reagents should be directed to and will be fulfilled by the lead contact, Kyunghwan Kim (kyungkim@chungbuk.ac.kr).

### Materials availability

This study did not generate new unique reagents.

### Data and code availability

• RNA-seq data have been deposited at GEO and are publicly available as of the date of publication. Accession numbers are listed in the key resources table.
• This article does not report the original code.
• All other data reported in this paper will be shared by the lead contact upon request.

## Acknowledgments

This work was carried out with the support of the National Research Foundation of Korea (2020R1A6A1A06046235, 2021RIS-001, and 2023R1A2C1006401 to K.K.; 2022R1I1A1A01069534 to S.J.Y.) and the Chungbuk National University BK21 program (2022).

## Author contributions

Conceptualization: J.P. and K.K.; methodology: J.P., H-Y.J., E.H.B., M.S., and J-I.P.; formal analysis: S.K. and S-Y.C.; investigation: J.P., S-J.Y., and K.K.; writing – original draft: J.P., S-J.Y., and K.K.; writing – review and editing: S-J.Y. and K.K.; supervision: K.K.

## Declaration of interests

The authors declare no competing interests.

## STAR★Methods

### Key resources table

**Table:** 

REAGENT or RESOURCE	SOURCE	IDENTIFIER
**Antibodies**		

Mouse monoclonal anti-NFATc1	Santa Cruz	Cat# sc-7294; RRID:AB_2152503
Rabbit monoclonal anti-Cathepsin K	Cell signaling	Cat#57056; RRID:AB_3096128
Mouse monoclonal anti-DC-STAMP	Millipore	Cat#MABF39-I; RRID:AB_10807703
Mouse monoclonal anti-β2 Tubulin	Santa Cruz	Cat#SC-47751; RRID:AB_2210539
Rabbit monoclonal anti-NFkB p65	Cell signaling technoloy	Cat# 8242; RRID:AB_3099658
Mouse monoclonal anti-β-Actin	Sino Biological	Cat# 100166-MM10; RRID:AB_2860060
Mouse monoclonal anti-LaminA/C	Santa Cruz	Cat# sc-376248; RRID:AB_10991536
Anti-TLR4	Protein Tech	Cat# 19811-1-AP; PR1D:AB_10638446
Rabbit polyclonal anti- NFkB p65	Diagenode	Cat# C15310256; RRID:AB_2721009 (ChIP)

**Chemicals, peptides, and recombinant proteins**		

M-CSF	This paper	N/A
RANKL	This paper	N/A
Endotoxin Removal resin	Thermofisher scientific	Cat# 88270
EZ-Cytox	DoGenBio	Cat# EZ-3000
Tri-RNA reagent	Favorgen	Cat# FATRR001
M-MLV Reverse transcriptase	Promega	Cat# M1708
iQ SYBR Green Supermix	Bio-rad	Cat# 1708882
TAK242	Sigmaaldrich	Cat# 614316
MyD inhibitor	InvivoGen	Cat# tlrl-pimyd

**Critical commercial assays**		

Acid phosphatase Leukocyte (TRAP) kit	Sigma-Aldrich	Cat# 386A
ATP Assay System Bioluminescence Detection Kit	Promega	Cat# FF2000

**Deposited data**		

RNA-seq data	This paper	GEO: GSE267039

**Experimental models: Cell lines**		

Mouse: WT ICR	Raonbio	
Mouse: WT C57BL/6J	Raonbio	
Mouse: *Prdx1−/−*	Gift from Dr. Dae-Yeul Yu (Ref)	N/A

**Oligonucleotides**		

Primers, see Table S3	This paper	N/A

**Recombinant DNA**		

pET15b-PRDX1 WT	This paper	N/A
pET15b-PRDX1 C83S	This paper	N/A
pET15b-PRDX1 4CS	This paper	N/A

**Software and algorithms**		

Prism 10	GraphPad software	RRID:SCR_002798
HOMER	http://homer.ucsd.edu/homer/	
Metascape	http://metascape.org	
TRRUST	https://www.grnpedia.org/trrust/	
Biorender	https://www.biorender.com/	
Bioluminate	https://www.schrodinger.com/platform/products/bioluminate/	

### Experimental model and subject details

#### Cell culture

Osteoclast precursor (OCP) cells were collected from femurs and tibias of 6-week-old male ICR mice and cultured in minimum essential medium- α (MEM-α) supplemented with 10% FBS and M-CSF (5 ng/mL) for 16 h. Nonadherent cells were collected and further cultured with M-CSF (30 ng/mL) for 3 days. After removal of floating cells, adherent cells were used as osteoclast precursor cells (OCP cells).

#### Mice

All mice were housed in mouse facilities at 12-h light/dark cycles in a 22°C temperature. All procedures were approved by the Institutional Animal Management and Use Committee of Chungbuk National University (CBNUA-1245-19-02). The mice were on a C57BL/6J or ICR background. Prdx1 knockout mice were kindly provided by Dr. Dae-Yeul Yu at Korea Reseach Institute of Bioscience and Biotechnology and were maintained in C57BL/6J background.^40^ Age-matched male mice were used for experiments.

### Method details

#### Construct and protein preparation of PRDX1

To generate the His6-tagged PRDX1 construct, the corresponding cDNA was ligated into the pET15b vector. For the PRDX1 mutants (C83S and 4CS), site-directed mutagenesis was performed to replace Cys52 with Ser52, Cys71 with Ser71, Cys83 with Ser83, and Cys153 with Ser153. Recombinant His6-tagged PRDX1 were expressed in *Escherichia coli* Rosetta2 and purified according to manufacturer’s protocol. Endotoxins were removed from all purified PRDX1 proteins using Endotoxin Removal resin (Thermofisher scientific).

#### Osteoclastogenesis and TRAP staining

OCP cells were grown on a 48-well plate with M-CSF (30 ng/mL) and RANKL (100 ng/mL) in MEM-α complete media. After 3–4 days, cells were fixed and stained for TRAP using an acid phosphatase leukocyte kit (Sigma-Aldrich). TRAP-positive multinucleated cells containing three or more nuclei are counted as osteoclasts under a light microscope.

#### Cell viability assay

OCP cells were seeded at a density of 5,000 cells/100 μL per well in 96-well plate and cultured with M-CSF (30 ng/mL) in MEM-α complete media. The viability of the cells was assessed daily using EZ-Cytox (DoGenBio).

#### RNA-sequencing

OCP cells were cultured with or without RANKL (100 ng/mL) in the presence or absence of PRDX1(200 nM). After a 2-day incubation, total RNA was isolated with Tri-RNA reagent (Favorgen, FATRR001). High-throughput sequencing with 100 bp pair-end was performed using NovaSeq 6000 (Illumina). The RNA-seq data was analyzed using the HOMER program, with cutoff parameters set at fold change ≥1.5 and FDR <0.05. Gene ontology (GO) analysis was performed using the Metascape tool (https://metascape.org/), and Gene Set Enrichment Analysis (GSEA) of MsigDB gene sets was conducted. TRRUST2 v2 (https://www.grnpedia.org/trrust/) was utilized for predicting transcription factors of target genes. RNA-seq data were available at NCBI Gene Expression Omnibus (GSE267039).

#### Immunoblotting

Whole cell lysates were prepared with SDS lysis buffer [20 mM Tris, pH7.5, 50 mM NaCl, 2.5% SDS, 2.5% sodium deoxycholate, protease inhibitor cocktails, and 0.5mM PMSF]. To isolate nuclear fractions, cells were lyzed with buffer A [10 mM HEPES pH7.9, 10 mM KCl, 1.5 mM MgCl_2_, 340 mM Sucrose, 10% Glycerol, 0.1% Triton X-100, 1 mM DTT, 0.4 mM PMSF and protease inhibitor cocktails]. Following centrifugation at 300*g* for 5 min, the nuclear pellet was further lysed with SDS lysis buffer. Subsequently, cell lysates were separated by SDS-PAGE and transferred onto PVDF membranes for immunoblotting using specific antibodies. The antibodies used included NFATc1 (Santa Cruz), NF-κB p65 (Cell Signaling), lamin A/C (Santa Cruz), and β-actin (Santa Cruz).

#### Measurement of ATP levels

ATP levels were quantified using a luciferin-luciferase based assay with the ENLITEN ATP Assay System Bioluminescence Detection Kit (Promega), following the manufacturer’s instructions. Briefly, OCP cells were seeded in 12-well plates with M-CSF (30 ng/mL) and RANKL (100 ng/mL) in MEM-α complete media, with or without PRDX1 (200 nM). After 1 day, cells were washed with PBS and lysed with the lysis buffer. Unnecessary components were precipitated using 2% trichloroacetic acid, and supernatants were neutralized by adding Tris-Acetate buffer. A 50 μL aliquot of luciferase reagent was then added to 50μL of the neutralized sample, and ATP levels were measured immediately using a luminometer.

#### Chromatin immunoprecipitation (ChIP)

ChIP was performed as previously described.^61^ Cells were crosslinked with 1% formaldehyde for 10 min and then washed twice with ice-cold PBS. The crosslinked cells were lysed using a hypotonic buffer [10 mM HEPES-KOH pH 7.8, 10 mM KCl, 1.5 mM MgCl_2_, and protease inhibitors] on ice for 10 min, followed by centrifugation at 14,000 rpm for 1 min. The resulting nuclear pellet was resuspended in a nuclear lysis buffer [1% SDS, 50 mM Tris-HCl pH 8.0, 10 mM EDTA]. Samples were sonicated for 20 cycles using a Bioruptor (Diagenode). Immunoprecipitation was performed on precleared chromatin preparations using antibodies specific for p65 (Diagenode) and IgG, along with A/G agarose beads. The precipitated DNA was subjected to real-time quantitative PCR with primers specific for the promoter regions of *Saa3*, *Acod1*, and *Nfatc1*. The PCR primer sequences for ChIP-qPCR can be found in Table S3.

#### Protein-protein docking simulation

3D models for monomer, dimer, and decamer PRDX1, as well as huTLR4, were generated using the BioLuminate module with existing structures as templates (PDB IDs: 3FXI, 2RII, and 7IH1). Protein-protein docking simulations were conducted with PIPER in BioLuminate, focusing on interactions between huTLR4 and mono-, di-, and deca-PRDX1. PIPER utilized FFT for a global search, selecting the top 30 clusters from 1000 conformations, with a 9 Å cluster radius. Each docked complex underwent 70,000 permitted rotations. The BioLuminate module calculated energies, defining the PIPER pose score to identify low-energy models. PIPER scores were determined for the best PRDX1-huTLR4 models.

#### Pull down assay

OCP cells were grown with M-CSF (30 ng/mL) in MEM-α complete media. Whole cell lysates were prepared using RIPA buffer [300 mM NaCl, 50 mM Tris-HCl pH 8.0, 0.5% deoxycholate, 0.1% SDS, 20 mM imidazole, 0.1% NP40, and protease inhibitor cocktails]. Recombinant wild-type PRDX1 or PRDX1 4CS mutant (10 μg) was then incubated with 1 mg of the lysate and Ni-NTA beads at 4°C overnight. Beads were washed three times with the same buffer, and the bound proteins were analyzed by SDS-PAGE, followed by immunoblotting using an anti-TLR4 antibody (ProteinTech).

#### Measurement of itaconate

Cells were washed with ice-cold 0.9% sterile NaCl, followed by addition of a solution of 80% (v/v) methanol and agitation for 10 min on ice. Intracellular metabolites were extracted by centrifugation at 21130*g* for 10 min, and the liquid supernatant was collected and filtered through a 0.2 μm PVDF membrane syringe filter. Samples were derivatized by incubating with a solution of 20 mg/mL methoxyamine hydrochloride in pyridine for 90 min at 30°C, followed by addition of N,O-bis(trimethylsilyl)trifluoroacetamide and heating for 30 min at 60°C. GC-MS analysis was conducted using a Thermo Trace 1310 GC coupled to a Thermo ISQ LT single quadrupole mass spectrometer, with separation on a DB-5MS column. The injection temperature was 300°C with a split ratio of 1:5. Metabolites were separated using a helium flow of 1.5 mL and the oven program included temperature ramps from 50°C to 325°C. Mass spectra were acquired in the scan range 35–650 m/z, processed using MS-DIAL (http://prime.psc.riken.jp/compms/msdial/main.html), and quantified based on standard calibration curves.

#### Real-time quantitative RT-PCR

Total RNA was prepared using the Tri-RNA reagent (Favorgen), and cDNA was generated using M-MLV reverse transcriptase (Promega) according to the manufacturer’s instructions. Real-time PCR was performed using the IQ SYBR Green Supermix and the IQ5 real-time cycler (Bio-Rad). Relative mRNA levels were normalized to β-actin mRNA levels. The primers used for RT-PCR are described in Table S3.

### Quantification and statistical analysis

Data are presented as mean ± SD. Statistical significance was assessed using a two-tailed t-test or one-way ANOVA followed by Tukey’s multiple comparison test for comparisons among three or more groups. A *p* value of <0.05 was considered significant. In the figures, asterisks denote statistical significance (∗*p* < 0.05; ∗∗*p* < 0.01; ∗∗∗*p* < 0.001; ∗∗∗∗*p* < 0.0001). Statistical analyses were performed using GraphPad Prism 10.

## Supplemental information

Document S1. Figures S1–S5 and Table S3

Table S1. K-means clustering of DEGs, related to Figure 2

Table S2. Transcriptomic changes in PRDX1 treatment, related to Figure 3
