# Supplementary material for: Peroxiredoxin 1-Toll-like receptor 4-p65 axis inhibits receptor activator of nuclear factor kappa-B ligand-mediated osteoclast differentiation
Source: iScience. 2024 Nov 26;27(12):111455. doi: 10.1016/j.isci.2024.111455 (PMC11667055; doi:10.1016/j.isci.2024.111455)
Supplement: Document S1. Figures S1–S5 and Table S3 [file mmc1.pdf]

iScience, Volume *27*

Supplemental information

Peroxiredoxin 1-Toll-like receptor 4-p65 axis inhibits receptor activator of nuclear factor kappa-B ligand-mediated osteoclast differentiation

Jisu Park, Sanggil Kim, Hye-Yeon Jung, Eun Hwan Bae, Minhye Shin, Jae-Il Park, So-Young Choi, Sun-Ju Yi, and Kyunghwan Kim

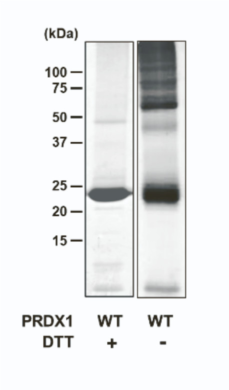

Figure S1. Coomassie staining of recombinant PRDX1 wild-type (WT), Related to Figure 1.

His-tagged PRDX1 was expressed in *E. coli*, and purified using Ni-NTA beads. Purified proteins were analyzed by SDS-PAGE under non-reducing (-DTT) and reducing (+DTT) conditions.

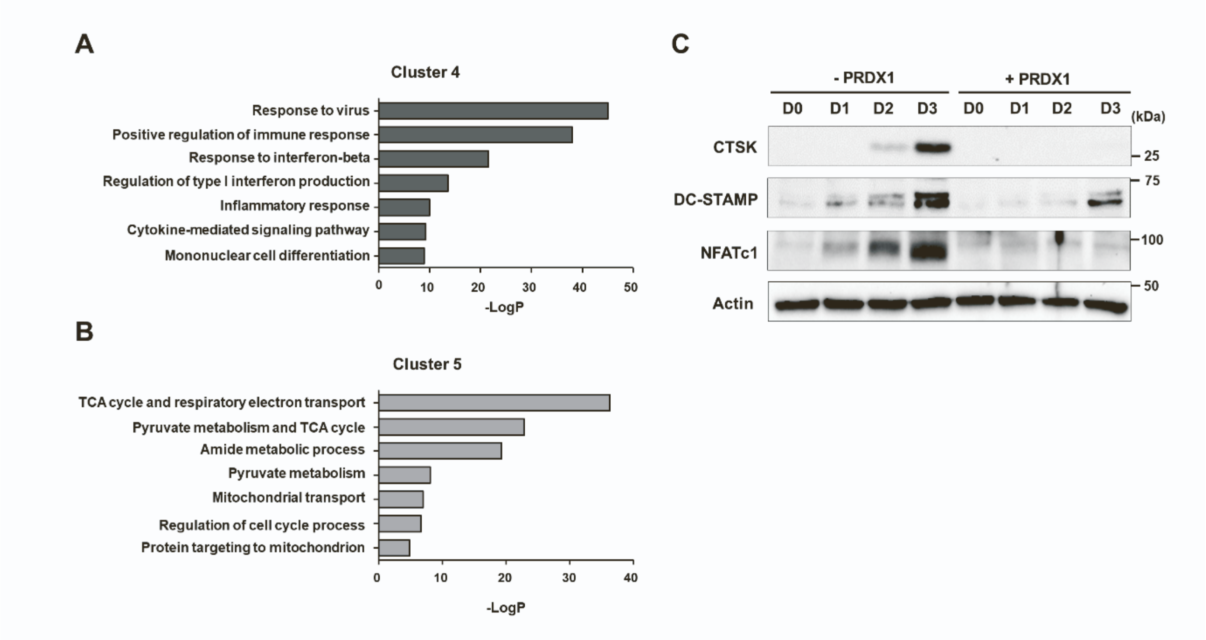

Figure S2. Gene profiling analysis of PRDX1 during osteoclastogenesis, Related to Figure 2.

(A and B) Gene ontology analysis for genes in clusters 4 and 5.

(C) An inhibitory effect of PRDX1 on the expression of osteoclast marker genes during osteoclast differentiation. OCP cells were cultured with or without PRDX1 (200 nM) in the presence of M-CSF (30 ng/ml) and RANKL (100 ng/ml). Whole-cell lysate were analyzed by immunoblotting with the indicated antibodies. β-Actin served as the loading control.

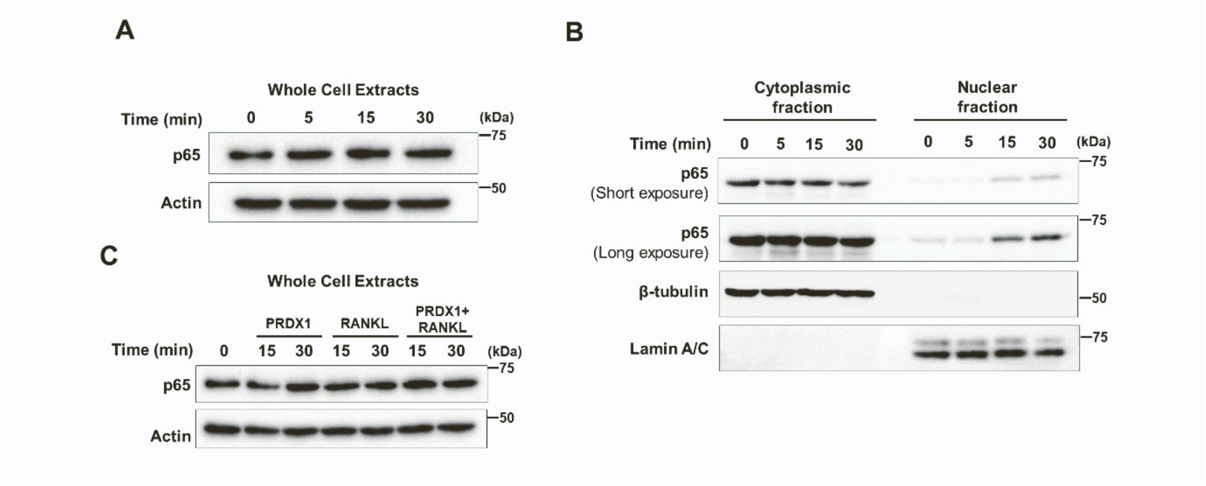

Figure S3. Effect of PRDX1 and RANKL on p65 expression levels, Related to Figure 4.

(A) OCP cells were treated with PRDX1 (200 nM) as the indicated times. Whole cell extracts were analyzed by immunoblotting with p65 antibody. β-Actin was used as a loading control.

(B) OCP cells as in (A) were prepared into cytoplasmic and nuclear fractions. Subcellular localization of p65 was analyzed by immunoblotting using the specified antibodies.

(C) OCP cells were treated with a combination of PRDX1 (200 nM) and RANKL (100 ng/ml) as indicated. Whole cell extracts were analyzed by immunoblotting for p65. β-Actin was used as a loading control.

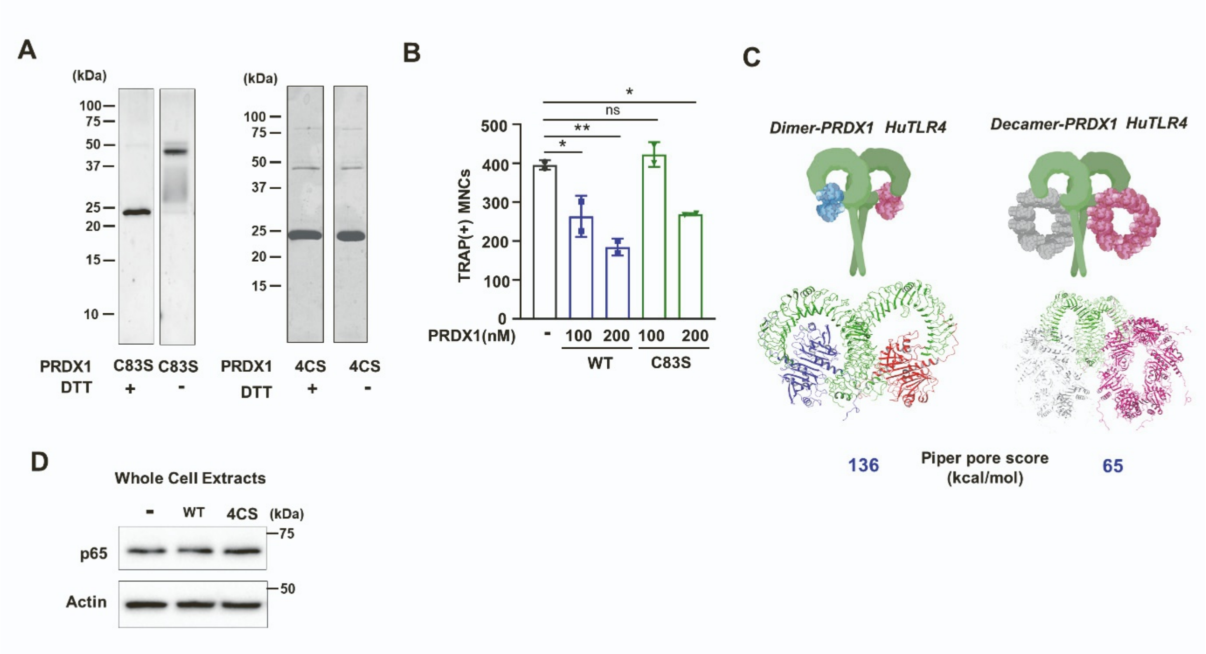

Figure 4. Effect of PRDX1 mutants on TLR4 signaling and osteoclast differentiation, Related to Figure 5.

(A) Coomassie staining of PRDX1 C83S and 4CS. Purified PRDX1 mutants were analyzed by SDS-PAGE under non-reducing (-DTT) and reducing (+DTT) conditions.

(B) TRAP staining of osteoclast precursors treated with PRDX1 WT or C83S. BMMs were cultured with varying concentrations of PRDX1 WT (100 and 200 nM) or PRDX1 C83S (100 and 200 nM) in the presence of M-CSF (30 ng/ml) and RANKL (100 ng/ml).

(C) Docking model between human TLR4 (HuTLR4) and PRDX1 mutants. The PIPER Pose Score was determined using the BioLuminate module.

(D) Effect of PRDX1 mutants on p65 expression levels. BMMs were treated with PRDX1 WT (100 nM) or 4CS mutant (25 nM) for 30min. Whole cell lysates were analyzed by immunoblotting with p65 antibody.

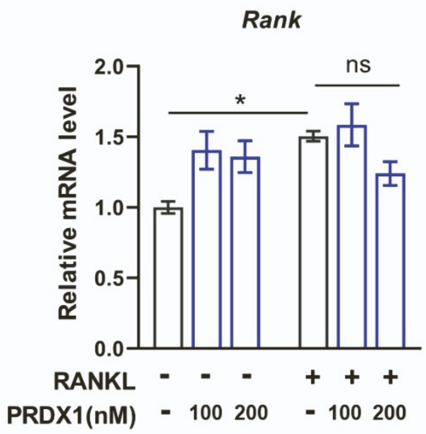

Figure S5. Effect of PRDX1 on *Rank* mRNA expression, Related to Discussion.

mRNA expression levels of *Rank* treated with PRDX1 (100 and 200 nM) and/or RANKL (100 ng/ml) for 1 day. The data were presented as Mean ± SD from three independent experiments. P value is determined by two-tailed t-test in (D) and one-way ANOVA with Tukey's multiple comparisons test. *P < 0.05; ns, not specified.

Table S3. Primers, Related to STAR Methods.

TARGET	Forward sequences (5’ → 3’)	Reverse sequences (5’ → 3’)
**RT-PCR**		
β-Actin	GCAAGTGCTTCTAGGCGGAC	AAGAAAGGGTGTAAAACGCAGC
Nfatc1	CTCGAAAGACAGCACTGGAGCAT	CGGCTGCCTTCCGTCTCATAG
Dcstamp	CCGCTGTGGACTATCTGCTG	CTCAATGGCTGCTTTGATCG
Ctsk	ACGGAGGCATTGACTCTGAAGATG-	GGAACCACCAACGAGAGGAGAAAT
Rank (Tnfrsf11a)	GCTGGCTACCACTGGAACTC	GTGCAGTTGGTCCAAGGTTT
Ccl5	CCTCACCATATGGCTCGGAC	TCTTCTCTGGGTTGGCACAC
Cfb	GTCAGGCCCTGGAGTACCTA	TCTTTTGGTCTCGGGTCTGC
Saa3	GGGTCTAGAGACATGTGGCG	TCCGGGCAGCATCATAGTTC
Acod1	TATGCCAACTACTCCCCCGA	GATGTGGTCAGCAGGGAACA
**ChIP**		
Saa3 (-1.9kb)	AACACCCCTGAGCTATTGCC	CCCACCCCTTTACGCAAAAG
Acod1 (-0.5kb)	TCCTTCTGGTAAGCAGGCTC	CAGAGGAAGAAAAGCCCCCA
Nfatc1 (-0.7kb)	CTCGTACAGCAAGCAATCCA-	ATGTAAAATCGCAGGCTTCC
